# Study on the safety and efficacy of CSP and HSP-EMR for small sessile colorectal polyps in the elderly population: Randomized controlled trial

**DOI:** 10.1097/MD.0000000000042863

**Published:** 2025-06-20

**Authors:** Hui-Hui Shang, Xiu-Mei Tian, Yan Li, Rong Zhang, Yu Wang, Wen-Xian Song

**Affiliations:** a Department of Health Medicine, The 991st Hospital of the Joint Logistics Support Force of the People's Liberation Army, Xiangyang, Hubei, China; b Department of Gastroenterology, The 991st Hospital of the Joint Logistics Support Force of the People's Liberation Army, Xiangyang, Hubei, China; c Department of Surgery, The 991st Hospital of the Joint Logistics Support Force of the People's Liberation Army, Xiangyang, Hubei, China; d Department of Pharmacy, The 991st Hospital of the Joint Logistics Support Force of the People's Liberation Army, Xiangyang, Hubei, China.

**Keywords:** cold snare resection, colorectal polyps, complication, hot snare polypectomy endoscopic mucosal resection, immediate bleeding, the elderly population

## Abstract

**Background:**

This study evaluates the safety and efficacy of cold snare polypectomy (CSP) and hot snare polypectomy endoscopic mucosal resection (HSP-EMR) for small sessile colorectal polyps in the elderly population.

**Methods:**

Patients with small sessile colorectal polyps were randomized to either CSP or HSP-EMR, including 80 patients in the observation group (CSP) and 125 patients in the control group (HSP-EMR). General data, titanium clip utilization rates, complete resection rates, immediate bleeding rates, specimen recovery rates, incidence of delayed bleeding, perforation rates, and incidence of abdominal discomfort were compared between the 2 groups.

**Results:**

There were no significant differences in the distribution of general data (age, gender, location, morphology, and pathological type) or underlying diseases (hypertension and diabetes) between the 2 groups (*P* > .05). There were no significant differences in the complete resection rate, specimen recovery rate, or delayed bleeding rate between the 2 groups (*P* > .05). The utilization rate of titanium clips and the incidence of abdominal discomfort in the observation group were significantly lower than those in the control group. The immediate bleeding rate was higher in the observation group than that in the control group. The difference between the 2 groups was statistically significant (*P* < .05). No perforation occurred in either of the groups. Group discussion was conducted according to whether postoperative complications occurred. Univariate and binary logistic regression analysis was used to analyze the risk factors of postoperative complications. The average polyp diameter, body mass index and surgical method were independent risk factors for postoperative complications (*P* < .05).

**Conclusion:**

CSP and HSP-EMR are safe and efficacy in the treatment of small sessile colorectal polyps in elderly people. For overweight and obese people with large polyps, HSP-EMR patients should be closely observed for postoperative complications.

## 1. Introduction

In 2020, the global incidence of new cases of colorectal cancer (CRC) was the 3rd highest among all malignant tumors, and the mortality rate was the 2nd highest,^[1]^ representing a significant threat to global health. The occurrence of the majority of CRC is closely associated with the presence of adenomatous polyps.^[2]^ The removal of adenomatous polyps in a timely manner has been demonstrated to be an effective strategy for reducing the incidence of CRC.^[3]^ CRC screening programs reduce CRC incidence,^[4]^ as the endoscopic removal of adenomas prevents their progression to CRC.^[5]^ Approximately 90% of colorectal polyps identified via colonoscopy are classified as small (diameter < 10 mm), of which 65% are adenomatous. Therefore, it is crucial to ensure safe and effective resection of small colorectal polyps. There are numerous endoscopic polypectomy techniques for the optimal treatment of small polyps (6–10 mm), including hot snare polypectomy endoscopic mucosal resection (HSP-EMR) and cold snare polypectomy (CSP). High frequency electrocoagulation included in HSP-EMR have been associated with an increased risk of postoperative bleeding and perforation due to thermal coagulation.^[6]^ So the recommendations set forth in the guidelines for individuals over the age of 50 years to undergo colonoscopy and endoscopic polypectomy, the relationship between adenomas and CRC in the elderly population is well-established.^[7]^ This study analyzed the clinical data of elderly patients who underwent CSP and HSP-EMR for small sessile colorectal polyps to identify the optimal treatment method for this population.

## 2. Materials and methods

### 2.1. Research object

This study was conducted in 991st Hospital of Joint Logistics Support Force of the People’s Liberation Army from December 2022 to October 2024. The patients included a total of 205 patients with small sessile colorectal polyps, 118 males and 87 females with an average age of 63.03 ± 8.60 years. The inclusion criteria were as follows: age between 50 and 80 years, regardless of gender; colon polyps with a diameter of 5 to 9 mm, classified as Paris type I or IIa; CSP and HSP-EMRs were selected; and with more than 1 eligible polyp, only the polyp treated 1st was included, and the rest were excluded. The exclusion criteria were as follows: patients who had taken antiplatelet drugs or anticoagulants in the previous week. The patient had inflammatory bowel disease, gastrointestinal polyposis, or malignant tumor. The patient had severe cardiopulmonary dysfunction. The patients were randomly divided into 2 groups: the observation group (CSP group, n = 80), and control group (HSP-EMR group, n = 125). There was no significant difference in the general data between the 2 groups (*P* > .05), indicating comparability.

### 2.2. Methods

#### 2.2.1. The intestine must be cleaned prior to treatment

A slag-free liquid diet was administered on the day preceding the procedure, and 2L of compound polyethylene glycol electrolyte powder was administered orally 4 to 6 hours before treatment. The powder was administered at a constant rate over 10 to 15 minutes, with the entire dose administered within 2 hours. It is also recommended that patients engage in exercise while taking medication to promote excretion. In the event of inadequate bowel preparation, such as constipation, bowel preparation should be performed 3 days in advance.

#### 2.2.2. The following outlines the treatment process

##### CSP procedure

 Colorectal polyps were identified during colonoscopy and their dimensions and morphology were accurately determined. narrow band imaging (NBI) was employed to observe glandular vascular morphology, thus ruling out the possibility of malignancy. The snare is placed, opened and placed 1 to 2 mm outside the normal mucosa of the polyp. Once the polyp was fully encompassed by the snare, the latter was retracted to dislodge the polyp, the resected polyps were collected by aspiration through the biopsy channel of the colonoscope.^[8]^ The surgical site was irrigated and NBI was employed to ascertain whether residual polyps, instances of immediate or active bleeding, and other pertinent details were present. A titanium clamp was used, if necessary.

##### HSP-EMR procedure

**Figure 1.**
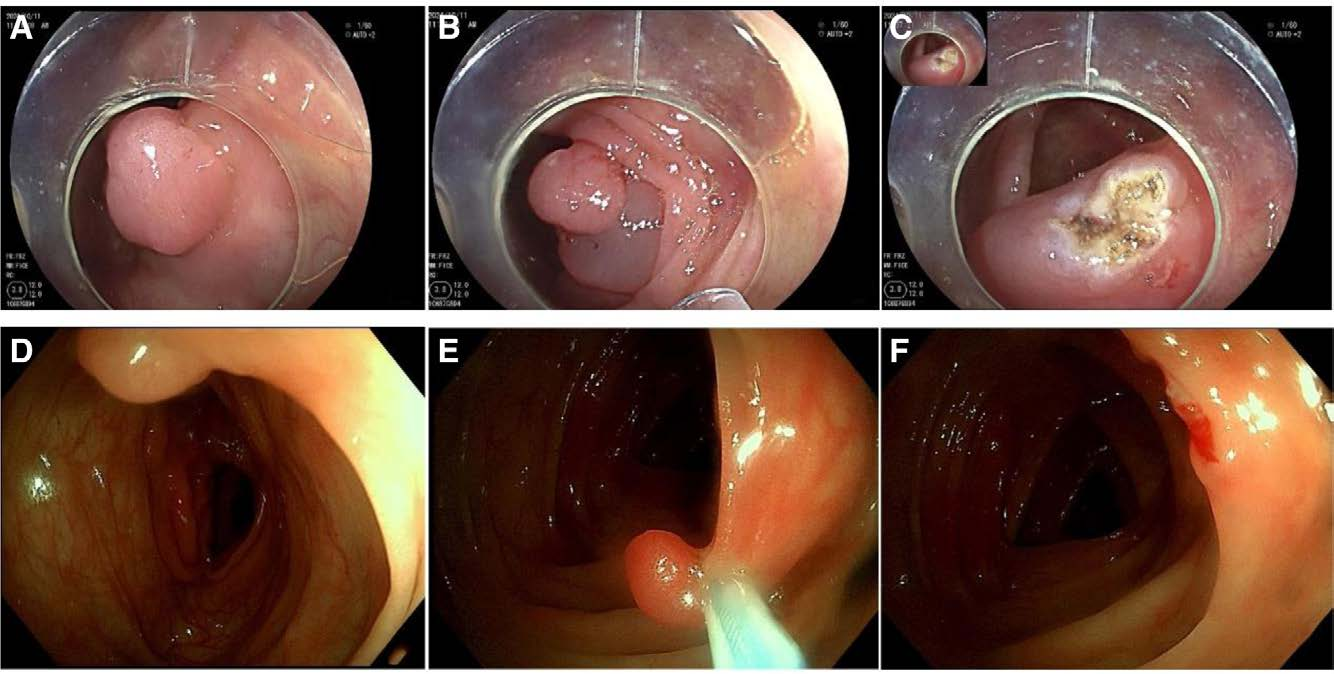
Endoscopic treatment images. (A) Images of colon polyps, (B) submucosal injection to lift the lesion, (C) HSP resection of the wound, (D) images of colon polyps, (E) CSP complete snare resection of polyps, (F) CSP resection of the wound. CSP = cold snare polypectomy, HSP= hot snare polypectomy.

 After NBI excluded malignant lesions, the lesion was lifted by submucosal injection, the snare was opened at the edge of the polyp, and the snare was gently lifted while being tightened. Subsequently, electrocution was performed for several seconds until the polyp was excised (Fig. 1). Samples were collected and subjected to pathological examination to ascertain the presence of immediate bleeding or active blood oozing. Following the procedure, the patient was advised to remain in bed and abstain from food on the day of the surgery. They were then fed a liquid diet for 2–3 days, gradually transitioned to a normal diet. During this period, the patient should be monitored for any systemic or abdominal symptoms including fever, abdominal pain, blood in the stool, and abdominal discomfort.

### 2.3. Observation target

The data were analyzed to ascertain the impact of age, body mass index (BMI), gender, and presence or absence of underlying diseases. A summary of the polyp location, morphology, and pathological type is provided. The surgical conditions of the 2 groups were analyzed, including the following main observation indicators: rates of complete resection, immediate bleeding (immediate bleeding was defined as bleeding which was not self-limiting within 2 minutes and required additional interventional hemostasis^[9]^), delayed bleeding (delayed bleeding was observed between 48 hours to 3 to 4 weeks following surgery^[10]^), abdominal discomfort, and perforation. The secondary outcome measures were titanium clip use, specimen recovery rate and operation time (from subject to the equipment being placed on the wound to stop bleeding and the collection of specimens).

### 2.4. The occurrence of postoperative complications

Postoperative complications were defined as delayed bleeding, postoperative abdominal discomfort and perforation. The general data and intraoperative conditions of the patients with postoperative complications were evaluated.

### 2.5. Statistical analysis

SPSS 22 software (Armonk) was used for statistical analysis. Continuous and categorical variables were presented as mean ± standard deviation (*x̄* ± *s*) and counts or percentages, using student *t* test or 1-way analysis of variance and chi-square test for comparisons between the 2 groups, respectively. The classification of data with significant comparison between groups was included in binary logistic regression to analyze the risk factors of postoperative complications. *P* < .05 was considered statistically significant.

## 3. Results

### 3.1. The general clinical characteristics of patients in both groups

No statistically significant differences were observed in the general data (age, gender, BMI, polyp diameter, location, morphology, and pathological type of polyps) between the 2 groups (*P* > .05). Similarly, no statistically significant differences were identified in the distribution of combined underlying diseases (hypertension and diabetes) between the 2 groups (*P* > .05), which was comparable (Table 1).

**Table 1:** General data of patients in the 2 groups [n (%), *x̄* ± *s*].

Variable		Observation group (CSP) n = 80 (%)	Control group (HSP-EMR) n = 125 (%)	*t*/*X*^2^	*P*
Gender				0.00	.99
	Male	46 (57.5)	72 (57.6)		
	Female	34 (42.5)	53 (42.4)		
Average age/yr		63.35 ± 9.61	62.83 ± 7.96	0.42	.68
BMI (kg/m^2^)		23.48 ± 1.77	23.75 ± 2.22	−0.94	.35
Hypertension				1.97	.16
	Yes	42 (52.5)	78 (62.4)		
	No	38 (47.5)	47 (37.6)		
Diabetes				1.20	.27
	Yes	9 (11.25)	21 (16.8)		
	No	71 (88.75)	104 (83.2)		
Location				0.24	.62
	Colon	72 (90)	115 (92)		
	Rectum	8 (10)	10 (8)		
Diameter (mm)		6.34 ± 0.83	6.34 ± 0.94	.01	.99
Morphology				2.08	.15
	Is	63 (78.75)	87 (69.6)		
	IIa	17 (21.25)	38 (30.4)		
Pathological type					
	Adenoma	68 (85)	108 (86.4)	0.08	.78
	Non-adenoma	12 (15)	17 (13.6)		

BMI = body mass index, CSP = cold snare polypectomy, HSP-EMR = hot snare polypectomy endoscopic mucosal resection.

### 3.2. Efficacy and safety evaluation indexes in 2 groups

Table 2 showed the efficacy and safety evaluation indexes in 2 groups. There were no significant differences in the complete resection rate, specimen recovery rate, or delayed bleeding rate between the 2 groups (*P* > .05). The immediate bleeding rate in the observation group was higher than that in the control group (12.5% vs 2.4%). The utilization rate of titanium clips in the observation group was significantly lower than that in the control group (7.5% vs 20%). The incidence of abdominal discomfort in observation group was significantly lower than that in control group (1.25% vs 20.8%). The operation time of the observation group was shorter than that of the control group (2.53 ± 0.52 minutes vs 5.02 ± 1.70 minutes), and these differences were statistically significant (*P* < .05). No perforation occurred in either the observation or the control group.

**Table 2:** Comparison of efficacy and safety indexes between the 2 groups [n (%),*x̄* ± *s*].

Variable		Observation group (CSP) n = 80 (%)	Control group (HSP-EMR) n = 125 (%)	*t*/*X*^2^	*P*
Titanium clip usage				5.94	.02
	Yes	6 (7.5)	25 (20)		
	No	74 (92.5)	100 (80)		
Complete resection				0.04	.84
	Yes	79 (98.75)	123 (98.4)		
	No	1 (1.25)	2 (1.6)		
Specimen recovery				.69	.41
	Yes	78 (97.5)	119 (95.2)		
	No	2 (2.5)	6 (4.8)		
Immediate bleeding				8.38	0
	Yes	10 (12.5)	3 (2.4)		
	No	70 (87.5)	122 (97.6)		
Delayed bleeding				2.61	.11
	Yes	0 (0)	4 (3.2)		
	No	80 (100)	121 (96.8)		
Perforation					
	Yes	0 (0)	0 (0)		
	No	100 (100)	100 (100)		
Abdominal discomfort				16.30	0
	Yes	1 (1.25)	26 (20.8)		
	No	79 (98.75)	99 (79.2)		
Operation time (min)		2.53 ± 0.52	5.02 ± 1.70	−12.74	0

CSP = cold snare polypectomy, HSP-EMR = hot snare polypectomy endoscopic mucosal resection.

### 3.3. Analysis of postoperative complications in 2 groups

Postoperative complications were determined by the presence of symptoms including abdominal discomfort, delayed bleeding and perforation. These were then divided into 2 groups: a complication group (n = 29) and a non-complication group (n = 176). Univariate analysis was performed on the average age, gender, BMI, polyp location, average diameter, morphology, pathological type, complete resection rate, immediate bleeding rate, titanium clip usage rate, operation time, and operation method of the 2 groups, respectively, showing statistically significant differences in BMI, polyp diameter, operation time, and operation method (*P* < .05, Table 3). Subsequent to this, the significant indexes were analyzed by single factor, and the insignificant variables were excluded to screen the related factors affecting postoperative complications. The binary logistic regression model (stepwise forward method) analysis, with or without complications as dependent variables and BMI, polyp diameter, operation time and operation method as independent variables, demonstrated that polyp diameter, operation time and BMI exhibited a positive correlation with postoperative complications, and the difference was statistically significant (*P* < .05, Table 4).

**Table 3:** Univariate analysis of postoperative complications [n (%), *x̄* ± *s*].

Variable		Complication group n = 29 (%)	No-complication group n = 176 (%)	*t*/*X*^2^	*P*
Gender				0.08	.78
	Male	16 (55.17)	102 (57.95)		
	Female	13 (44.83)	74 (42.05)		
Average age/yr		61.76 ± 7.31	63.24 ± 8.82	0.86	.39
BMI (kg/m^2^)		25.41 ± 2.19	23.35 ± 1.89	−5.31	0
Diameter (mm)		7.31 ± 0.93	6.18 ± 0.78	−7.03	0
Operation time (min)		4.87 ± 1.79	3.91 ± 1.81	−2.66	.01
Location	Colon	28 (96.55)	159 (90.34)	1.20	.27
	Rectum	1 (3.45)	17 (9.66)		
Morphology	Is型	19 (65.52)	131 (74.43)	1.01	.32
	IIa型	10 (34.48)	45 (25.57)		
Pathological type	Adenoma	27 (93.10)	149 (84.66)	1.46	.23
	Non-adenoma	2 (6.90)	27 (15.34)		
Immediate bleeding	Yes	4 (13.79)	9 (5.11)	3.16	.08
	No	25 (86.21)	167 (94.89)		
Titanium clip usage	Yes	8 (27.59)	23 (13.07)	4.09	.04
	No	21 (72.41)	153 (86.93)		
Complete resection	Yes	27 (93.10)	175 (99.43)	6.92	.01
	No	2 (6.90)	1 (0.57)		
Operation type	CSP	1 (3.45)	79 (44.89)	17.97	0
	HSP-EMR	28 (96.55)	97 (55.11)		

BMI = body mass index, CSP = cold snare polypectomy, HSP-EMR = hot snare polypectomy endoscopic mucosal resection.

**Table 4:** Multivariate analysis of postoperative complications.

Variable	β	SE	Waldχ2	OR	95% CI	*P*
Diameter	1.750	0.376	21.617	5.753	2.751–12.028	.000
HSP-EMR	3.638	1.281	8.057	37.997	3.083–468.342	.005
BMI	0.437	0.147	8.834	1.549	1.161–2.066	.003

BMI = body mass index, CI = confidence intervals, HSP-EMR = hot snarepolypectomy endoscopic mucosal resection, OR = odds ratios, SE = standard error.

## 4. Discussion

Colorectal polyps are swelling of the colorectal mucosa, and are closely related to dietary habits, genetics, inflammation, foreign bodies, and other factors.^[11]^ The Chinese guidelines for cancer prevention and control suggest that all polyps should be removed, except those confined to the sigmoid colon and rectum with a diameter of <5 mm.^[12]^ The endoscopic resection of small colorectal polyps is straightforward, convenient, and safe. The most appropriate treatment method was selected based on polyp size and pathological type. For intestinal polyps with a diameter of ≤5 mm, biopsy forceps may be used for direct removal. Intestinal polyps with a diameter of ≥10 mm can be removed using HSP or EMR. The optimal removal method for intestinal polyps with diameters of 5 to 10 mm remains debatable.^[13]^ The use of hot biopsy forceps necessitates electrocoagulation and cauterization, which increase the risk of complex perforation and delayed bleeding. The use of electrocoagulation cauterization of specimens during surgery presents certain difficulties in postoperative pathological judgement and is therefore rarely employed in current practice.^[14]^ Resection of non-treated polyps <10 mm in diameter by CSP has been demonstrated to achieve a high complete resection rate (93.5–98.2%), a low incidence of intraoperative immediate bleeding (0–7.8%), a low rate of postoperative delayed bleeding (0–0.5%), and an almost negligible risk of perforation.^[15,16]^ Therefore, the European guidelines suggest that CSP should be used for resection of small colorectal polyps.^[17]^ China guideline recommendations that CSP should be used for the removal of non-pediculated colorectal polyps measuring <10 mm in diameter.^[18]^

Complete resection is an essential indicator for assessing polypectomy efficacy. It has been demonstrated that incomplete resection of colorectal polyps represents a significant etiological factor in CRC, with an estimated range of 19.0% to 30.8%.^[19]^ Ito et al^[20]^ reported the complete resection rate of CSP was very high (100%), the R0 rate was not satisfactory (horizontal margin, 65.5%; vertical margin, 89.1%), CSP was safer than HSP. Suzuki et al^[21]^ reported the use of CSP for the removal of rectal and sigmoid polyps with a maximum diameter of 10 mm. The immediate postoperative wound width was significantly larger than that of HSP; however, the surgical wound size was much smaller than that of HSP after 1 day. The primary cause of wound expansion on the 1st postoperative day after HSP surgery was electrocoagulation of the mucosal tissue near the incisal margin, resulting in necrosis, which subsequently spread to the surrounding tissues. Furthermore, the mucosal muscle layer was included in the postoperative CSP resection specimens. A meta-analysis confirmed that there was no significant difference between CSP and HSP in incomplete resection rate, monolithic resection rate, and polyp recovery rate. In terms of safety, there were no significant differences between CSP and HSP in the rate of intraoperative bleeding and the rate of delayed bleeding per polyp. The total operation time of CSP was significantly shorter than that of HSP (*P* < .00001). Therefore, CSP is an effective and safe method to remove small colorectal polyps and can be used as a suitable alternative to HSP.^[22]^ Consequently, CSP can achieve the requisite width and depth of resection for diminutive colorectal polyps, thereby fulfilling the need for a complete resection. The latest study compares underwater CSP with conventional CSP for colorectal polyps 5 to 10 mm found that underwater cold snare polypectomy was superior to conventional cold snare polypectomy in mucosal muscle resection rate, with higher overall resection rate and lower incomplete resection rate, and R0 resection rate was not affected by the experience of endoscopists.^[23]^

Compared with traditional electropolypectomy, CSP has the advantage of reducing the need for submucosal injection and electrocoagulation. Furthermore, CSP has the additional advantages of convenient operation, low bleeding and perforation rates, and the capacity to inhibit the expression of serum prostaglandin E2, nerve growth factor, thereby promoting pain relief and reducing blood loss and polypectomy time.^[24]^ Nevertheless, it should be noted that wound treatment in the absence of an electrothermal effect may result in wound bleeding following CSP resection. Most wounds cease bleeding within a few seconds to minutes; however, some wounds continue to bleed. Aoki et al^^[25]^^ observed that, protruded lesion as an independent risk factor for immediate bleeding. The identification of high-risk factors associated with intraoperative bleeding, which could necessitate additional hemostatic procedures, has the potential to enhance the safety of CSP.

Delayed bleeding following CSP is uncommon in patients undergoing antithrombotic therapy (ATT) and does not result in major bleeding. In HSP treatment, ATT represents a risk factor for delayed bleeding irrespective of whether anticoagulant drugs are discontinued in advance. Additionally, the presence of large polyps and use of titanium clips are significant risk factors for postoperative bleeding.^[26]^ In patients with continuous anticoagulation, CSP has a low risk of bleeding and there is no need to interrupt anticoagulation therapy.^[27]^ The safety of HSP with low-power pure-cut current was comparable to that of CSP for nonpedunculated polyps <10 mm.^[28]^ Although CSP is safe and effective, attention should also be paid to the occurrence of adverse reactions. Kodama et al reported a case of 0-Is type lesions of 3 mm transverse colon undergoing intestinal wall hematoma after CSP.^[29]^

The results of this study indicated that there were no statistically significant differences in the complete resection rate, specimen recovery rate, and delayed bleeding rate between the observation group (CSP group) and the control group (HSP-EMR group; *P* > .05). The immediate intraoperative bleeding rates were 12.5% in the observation group and 2.4% in the control groups (*P* < .05). The findings indicated that for colorectal polyps measuring <10 mm, both CSP and HSP-EMR demonstrated favorable outcomes in terms of complete resection and specimen recovery. However, the incidence of immediate intraoperative bleeding was marginally higher in the CSP group, which was attributable to polyp removal by tightening with a snare instead of electrocoagulation. Nevertheless, immediate bleeding is due to the superficial submucosal/lamina propria excision that results from CSP whereby resultant bleeding is often from superficial capillaries rather than larger submucosal vessels.^[30]^ Studies have confirmed that the risk of immediate bleeding can be predicted by the size and shape of polyps and the size and shape of iatrogenic ulcers.^[31]^ During the operation, part of the drug was sprayed to achieve the hemostatic effect, and part of the titanium clip was applied. A meta-analysis confirmed that styptic powder has a significant effect on instant hemostasis of gastrointestinal bleeding, and both monotherapy and combination therapy are effective, which is comparable to traditional endoscopic therapy with high safety.^[32]^

The electrocoagulation technique was not employed during the CSP operation, thereby avoiding any heat damage and minimizing trauma to the body. This approach proved beneficial in terms of postoperative recovery. The incidence of abdominal discomfort in the observation group was 1% (1 case) lower than that in the control group (20.8%; 26 cases; *P* < .05). The absence of electrocoagulation in CSP reduces damage to the surrounding tissues. The use of electrocoagulation via hot snare resection not only results in the generation of heat and smoke but also causes significant damage to surrounding tissues. This can lead to complications such as postoperative abdominal pain and discomfort, and may also result in slower postoperative recovery. Previous studies have confirmed that the incidence of postoperative abdominal pain in the HSP group is higher than that in the CSP group, which is considered to be related to electrocoagulation.^[33]^ de Benito Sanz et al^[15]^ observed that, it was no differences in complete resection and bleeding rates between CSP and HSP. But CSP reduced the intensity and duration of post-colonoscopy abdominal pain.

Patients were divided into complication and non-complication groups according to postoperative abdominal discomfort, delayed bleeding and perforation. Univariate analysis showed that BMI, polyp diameter, operation time, operation method, complete resection and titanium stapler were associated with postoperative complications. Univariate analysis was significantly included in binary logistic regression analysis. Nonsignificant variables were removed by the forward stepwise method and it was found that BMI, polyp diameter and HSP-EMR were positively correlated with postoperative complications with statistical significance (*P*<.05). The reason for this is that overweight and obese people have more abdominal fat, and the intestinal peristalsis function itself is poor, and the operation further aggravates the intestinal dysfunction. The larger the diameter of the polyp, the greater the risk of delayed bleeding, abdominal pain and bloating. Univariate analysis suggested that operative time was associated with postoperative complications, but the use of a carbon dioxide pump in this study had little effect on postoperative negative abdominal distension and was considered a confounding factor. Therefore, both HSP-EMR and CSP are safe and effective methods for the treatment of 5 to 9 mm non-tuberous colorectal polyps in the elderly population, but CSP may reduce the occurrence of postoperative complications such as delayed bleeding and abdominal discomfort. For overweight and obese people, polyps with large diameter, the selection of HSP-EMR should pay attention to the occurrence of postoperative complications.

## 5. Limitations

In this study, complete resection was determined based on NBI staining and biopsies at the edge and bottom of the wound were not performed to ascertain the presence of residual tissue. This study did not include patients with high-risk factors who were unable to cease anticoagulant or antiplatelet medications. The patients are still in the postoperative follow-up period, and postoperative recurrence will report later. However, the sample size was insufficient to draw any definitive conclusions. Further investigations are required to compare the efficacy and safety of CSP-EMR and CSP in larger cohorts.

## 6. Conclusions

In conclusion, CSP has a high complete resection rate for small sessile colorectal polyps in the elderly population. Furthermore, compared to HSP-EMR, the utilization rate of titanium clips and the incidence of postoperative abdominal discomfort are lower, resulting in time and economic benefits, superior experience, and a safe and effective procedure. Therefore, CSP is worthy of further clinical application. For overweight and obese people with large polyps, HSP-EMR patients should be closely observed for postoperative complications.

## Acknowledgments

We would like to thank the participants for their cooperation and support.

## Author contributions

**Conceptualization:** Hui-Hui Shang, Wen-Xian Song.

**Data curation:** Xiu-Mei Tian.

**Formal analysis:** Xiu-Mei Tian, Yan Li, Rong Zhang, Yu Wang.

**Funding acquisition:** Wen-Xian Song.

**Methodology:** Wen-Xian Song.

**Project administration:** Wen-Xian Song.

**Resources:** Yu Wang.

**Writing – original draft:** Hui-Hui Shang.

**Writing – review & editing:** Hui-Hui Shang.

## References

[1] Sung H Ferlay J Siegel RL. Global cancer statistics 2020: GLOBOCAN estimates of incidence and mortality worldwide for 36 cancers in 185 countries. CA Cancer J Clin. 202l;71:209–49. 10.3322/caac.21660 33538338

[2] Sninsky JA Shore BM Lupu GV Crockett SD. Risk factors for colorectal polyps and cancer. Gastrointest Endosc Clin N Am. 2022;32:195–213. 35361331 10.1016/j.giec.2021.12.008

[3] Ikematsu H Murano T Shinmura K. Detection of colorectal lesions during colonoscopy. DEN Open. 2021;2:e68. 35310752 10.1002/deo2.68 PMC8828173

[4] Ransohoff DF. Colon cancer screening in 2005: status and challenges. Gastroenterology. 2005;128:1685–95. 15887159 10.1053/j.gastro.2005.04.005

[5] He X Hang D Wu K. Long-term risk of colorectal cancer after removal of conventional adenomas and serrated polyps. Gastroenterology. 2020;158:852–61.e4. 31302144 10.1053/j.gastro.2019.06.039 PMC6954345

[6] Takamaru H Saito Y Hammoud GM. Comparison of postpolypectomy bleeding events between cold snare polypectomy and hot snare polypectomy for small colorectal lesions: a large-scale propensity score-matched analysis. Gastrointest Endosc. 2022;95:982–9.e6. 34971668 10.1016/j.gie.2021.12.017

[7] Guo S Gu J Zhang D Wang X Li S. The elderly harbor greater proportions of advanced histology in subcentimeter adenomas: implications for screening colonoscopy approaches. Eur J Gastroenterol Hepatol. 2022;34:281–7. 34593701 10.1097/MEG.0000000000002284

[8] Horii T Suzuki S Sugita A. Comparison of complete resection rates in cold snare polypectomy using two different wire diameter snares: a randomized controlled study. J Gastroenterol Hepatol. 2023;38:752–60. 36565225 10.1111/jgh.16092

[9] Ferlitsch M Hassan C Bisschops R. Colorectal polypectomy and endoscopic mucosal resection: European Society of Gastrointestinal Endoscopy (ESGE) Guideline - Update 2024. Endoscopy. 2024;56:516–45. 38670139 10.1055/a-2304-3219

[10] Burgess NG Metz AJ Williams SJ. Risk factors for intraprocedural and clinically significant delayed bleeding after wide-field endoscopic mucosal resection of large colonic lesions. Clin Gastroenterol Hepatol. 2014;12:651–61.e1-3. 24090728 10.1016/j.cgh.2013.09.049

[11] Dong J Ma TS Xu YH. Characteristics and potential malignancy of colorectal juvenile polyps in adults: a single-center retrospective study in China. BMC Gastroenterol. 2022;22:75. 35189824 10.1186/s12876-022-02151-x PMC8862221

[12] National Clinical Research Center for Digestive Diseases (Shanghai), Chinese Society of Digestive Endoscopology, Cancer Endoscopy Professional Committee of China Anti-Cancer Association. Expert consensus on management strategies for precancerous lesions and conditions of colorectal cancer in China (In Chinese). Chin J Dig Endosc. 2022;39:1–18.

[13] Takeuchi Y Shichijo S Uedo N. Safety and efficacy of cold versus hot snare polypectomy including colorectal polyps ≥ lcm in size. Dig Endosc. 2022;34:274–83. 34324730 10.1111/den.14096

[14] Golda T Lazzara C Sorribas M. Combined endoscopic-laparoscopic surgery (CELS) can avoid segmental colectomy in endoscopically unremovable colonic polyps: a cohort study over 10 years. Surg Endosc. 2022;36:196–205. 33439344 10.1007/s00464-020-08255-3

[15] de Benito Sanz M Hernández L Garcia Martinez MI. Efficacy and safety of cold versus hot snare polypectomy for small (5-9mm) colorectal polyps: a multicenter randomized controlled trial. Endoscopy. 2022;54:35–44. 33264811 10.1055/a-1327-8357

[16] Chang LC Chang CY Chen CY. Cold versus hot snare polypectomy for small colorectal polyps: a pragmatic randomized controlled trial. Ann Intern Med. 2023;176:311–9. 36802753 10.7326/M22-2189

[17] Ferlitsch M Moss A Hassan C. Colorectal polypectomy and endoscopic mucosal resection (EMR): European Society of Gastrointestinal Endoscopy (ESGE) Clinical Guideline. Endoscopy. 2017;49:270–97. 28212588 10.1055/s-0043-102569

[18] Chinese Society of Digestive Endoscopy. Chinese expert consensus on cold snare polypectomy for colorectal polyps (2023, Hangzhou) (In Chinese). Chin J Gastrointest Endosc Electron Ed. 2023;10:73–82.

[19] Kang DK Park SB Kim HW. Long-term outcomes and surveillance timing of patients with large non-pedunculated colorectal polyps with histologically incomplete resection in endoscopic resection. Surg Endosc. 2022;36:1369–78. 33689013 10.1007/s00464-021-08419-9

[20] Ito T Takahashi K Tanabe H. Safety and efficacy of cold snare polypectomy for small colorectal polyps: a prospective randomized control trial and one-year follow-up study. Medicine (Baltim). 2021;100:e26296. 10.1097/MD.0000000000026296 PMC8202597 34115035

[21] Suzuki S Gotoda T Kusano C. Width and depth of resection for small colorectal polyps: hot versus cold snare polypectomy. Gastrointest Endosc. 2018;87:1095–103. 29122600 10.1016/j.gie.2017.10.041

[22] Winston K Maulahela H Raharjo D. A comparative analysis of the efficacy and safety of hot snare polypectomy and cold snare polypectomy for removing small colorectal polyps: a systematic review and meta-analysis. Cureus. 2023;15:e38713. 37292560 10.7759/cureus.38713 PMC10246601

[23] Zachou M Nifora M Androutsakos T. Results of the COLDWATER randomized controlled trial: enhanced performance of underwater cold snare polypectomy for colorectal polyps 5-10 mm, independent of endoscopist experience. Ann Gastroenterol. 2024;37:466–75. 38974083 10.20524/aog.2024.0889 PMC11226736

[24] Yuan CY Liu JN Zhong RM. Effects of endoscopic cold resection of colon polyps on blood loss and serum pain factor levels in patients with colon polyps (In Chinese). J Clin Exp Med. 2022;21:1057–60.

[25] Aoki T Kolligs FT Yoshida S, . Analysis of predictive factors for R0 resection and immediate bleeding of cold snare polypectomy in colonoscopy. PLoS One. 2019;14:e0213281. 30822318 10.1371/journal.pone.0213281 PMC6396914

[26] Aizawa M Utano K Nemoto D. Risk of delayed bleeding after cold snare polypectomy in patients with antithrombotic therapy. Dig Dis Sci. 2022;67:1869–78. 33973083 10.1007/s10620-021-06984-6

[27] Ferdinande K Desomer L De Looze D Tate DJ. Colonic polypectomy in 2024: hot or cold? Acta Gastroenterol Belg. 2024;87:505–16. 39745037 10.51821/87.4.13199

[28] Kimura H Oi M Imai K. Safety and efficacy of low-power pure-cut hot snare polypectomy for small nonpedunculated colorectal polyps compared with conventional resection methods: a propensity score matching analysis. DEN Open. 2024;5:e378. 38715897 10.1002/deo2.378 PMC11075073

[29] Kodama Y Mizokami Y Toyama Y. A case of gastrointestinal perforation following transarterial embolization for an intramural hematoma after cold snare polypectomy of an adenoma in the transverse colon. DEN Open. 2024;5:e70017. 39351043 10.1002/deo2.70017 PMC11439986

[30] Qu J Jian H Li L. Effectiveness and safety of cold versus hot snare polypectomy: a meta-analysis. J Gastroenterol Hepatol. 2019;34:49–58. 30176072 10.1111/jgh.14464

[31] Oh SJ Jung Y Hwangbo Y Cho YS Chung IK Lee CK. Prediction of immediate bleeding after cold snare polypectomy: a prospective observational study. Medicine (Baltim). 2024;103:e39597. 10.1097/MD.0000000000039597 PMC11383725 39252235

[32] Facciorusso A Straus Takahashi M Eyileten Postula C Buccino VR Muscatiello N. Efficacy of hemostatic powders in upper gastrointestinal bleeding: a systematic review and meta-analysis. Dig Liver Dis. 2019;51:1633–40. 31401022 10.1016/j.dld.2019.07.001

[33] Ichise Y Horiuchi A Nakayama Y Tanaka N. Prospective random comparison of cold snail resection polyps with traditional polyps in small colorectal polyps. Digestion.2011;84:78–81. 21494037 10.1159/000323959

