# Study on the safety and efficacy of CSP and HSP-EMR for small sessile colorectal polyps in the elderly population: A randomized controlled trial: Erratum

**DOI:** 10.1097/MD.0000000000043687

**Published:** 2025-07-18

**Authors:** Hui-Hui Shang, Xiu-Mei Tian, Yan Li, Rong Zhang, Yu Wang, Wen-Xian Song

In the article “Study on the safety and efficacy of CSP and HSP-EMR for small sessile colorectal polyps in the elderly population: A randomized controlled trial,”^[1]^ which appears in Volume 104, Issue 25 of *Medicine,* the title of the article was incorrectly published as “Study on the safety and effectiveness of CSP and HSP-EMR in small polyps colorectal cancer in the elderly population: Randomized controlled trial” when the issue was first posted on June 20, 2025, due to an error during the production process. The title has now been corrected in the online version.
